# Spark Plasma Sintered B_4_C—Structural, Thermal, Electrical and Mechanical Properties

**DOI:** 10.3390/ma13071612

**Published:** 2020-04-01

**Authors:** Ruslan Kuliiev, Nina Orlovskaya, Holden Hyer, Yongho Sohn, Mykola Lugovy, DongGi Ha, Miladin Radovic, Elinor G. Castle, Michael John Reece, Pradeep Vallachira Warriam Sasikumar, Laura Conti, Thomas Graule, Jakob Kuebler, Gurdial Blugan

**Affiliations:** 1 Department of Mechanical and Aerospace Engineering, University of Central Florida, Orlando, FL 32816, USA; Ruslan_Kuliiev@knights.ucf.edu (R.K.); Nina.Orlovskaya@ucf.edu (N.O.); 2 Renewable Energy and Chemical Transportation Cluster, University of Central Florida, Orlando, FL 32816, USA; 3 Department of Material Science and Engineering, University of Central Florida, Orlando, FL 32816, USA; hhyer@Knights.ucf.edu (H.H.); Yongho.Sohn@ucf.edu (Y.S.); 4 Institute for Problems of Materials Science, 3 Krzhizhanovsky St., 03142 Kyiv, Ukraine; nil2903@gmail.com; 5 Department of Material Science and Engineering, Texas A&M University, College Station, TX 77843, USA; hdk9212@gmail.com (D.H.); mradovic@tamu.edu (M.R.); 6 Centre for Materials Research, School of Engineering and Materials Science, Queen Mary University of London, Mile End Road, London E1 4NS, UK; e.g.castle@qmul.ac.uk (E.G.C.); m.j.reece@qmul.ac.uk (M.J.R.); 7 Laboratory for High-Performance Ceramics, Empa Swiss Federal Laboratories for Materials Science & Technology CH-8600 Dübendorf, Switzerland; Pradeep.vallachira-warriam@empa.ch (P.V.W.S.); Laura.conti@empa.ch (L.C.); thomas.graule@empa.ch (T.G.); jakob.kuebler@gmail.com (J.K.)

**Keywords:** boron carbide, mechanical properties, ring-on-ring test, finite elements analysis

## Abstract

The structural, thermal, electrical and mechanical properties of fully dense B_4_C ceramics, sintered using Spark Plasma Sintering (SPS), were studied and compared to the properties of B_4_C ceramics previously published in the literature. New results on B_4_C’s mechanical responses were obtained by nanoindentation and ring-on-ring biaxial strength testing. The findings contribute to a more complete knowledge of the properties of B_4_C ceramics, an important material in many industrial applications.

## 1. Introduction

Boron carbide (B_4_C) is an important ceramic material and plays a significant role in many industrial applications, such as abrasives, materials for nuclear plants or protective materials for ballistic impact. The choice of B_4_C in numerous applications is determined by its unique properties, such as low density, high hardness and elastic modulus, high neutron capture cross section and others [1]. Its density is in the range of 2.46 g/cm^3^ for B_10.4_C to 2.52 g/cm^3^ for B_4_C and is determined by the light elements that form B_4_C’s crystal structure increasing linearly with increasing carbon content where slightly different stoichiometry is defined by either excess B or C [2,3,4]. The crystal structure of B_4_C consists of 12-atom icosahedra units located at the vertices of a rhombohedra lattice, with 3-atom linear chains that link the icosahedra along the rhombohedral axis [5,6,7,8,9]. The reported rhombohedral lattice parameters of B_4_C are a = 5.16 Å and α = 65.7 Å, or, if represented as a hexagonal lattice, its lattice parameters become a_0_ = 5.60 Å, c_0_ = 12.07 Å with an axial ratio of c_0_/ a_0_ = 2.155 [10,11,12,13,14]. The $R\bar{3}m$ B_4_C structure implies the existence of four types of the atomic bonding—the interchain bonds, the chain icosahedral bonds, the intericosahedral bonds and the highly delocalized intraicosahedral sp2 bonds [5]; and their localization and delocalization, iconicity and covalent character along with the electron density determine the properties of B_4_C [15,16,17,18,19]. Group theory predicts 5*A_1g_* + 2*A_1u_* + 2*A_2g_* + 6*A_2u_* + 7*E_g_* + 8*E_u_* representation for the normal modes of lattice dynamics of $R\bar{3}m$ rhombohedral B_4_C, where the 12 modes of A_1g_ and Eg symmetry are Raman active, the 14 modes of A_2u_ and E_u_ symmetry are Infrared (IR) active and the A_1u_ and the A_2g_ modes are optically inactive. When the zero-frequency modes are removed, the number of IR active modes become 12 [20]. If carbon atoms are introduced into the icosahedron, a higher number of vibrational modes can be expected in the collected spectra [5]. 

It was reported that B_4_C exhibits a strong anisotropy of the elastic constants, meaning that elastic moduli are orientation dependent [21]. It was found that the Young’s modulus of B_5.6_C single crystal has a maximum E_max_ = 522 GPa along the [111] direction and a minimum Emin = 64 GPa and a maximum shear modulus Gmax = 233 GPa along the [201] direction and Gmin = 165 GPa in the [112] direction [5]. The elastic moduli of isotropic polycrystalline B_4_C have a dependence on the stoichiometry of the B or C atoms [22,23,24,25,26]. While B_4_C exhibits high elastic moduli, determined by the atomic bonding in the compound, the extremely rigid framework arising from the covalently bonded icosahedra and chain units leads to their refractory nature and extreme hardness [27]. B_4_C shows good flexure strength, which is dependent on external factors, such as processing conditions, size of the samples tested, grain size, porosity, presence of defects and so forth. Because B_4_C ceramic is relatively brittle, it shows a strong flaw sensitivity, where defects play a detrimental role and often are responsible for premature failure. Therefore, there has been a significant effort to improve the compaction technologies to provide enhancement in sintering, with the majority of the techniques used for densification of B_4_C being pressureless sintering [28,29,30,31,32], hot pressing [33,34,35,36], hot isostatic pressing [37], direct current sintering [38,39,40,41,42], high pressure sintering in multi-anvil apparatus [43] and contactless flash sintering [44] techniques. Currently, the most accepted and well developed processing technique for densification of B_4_C is the direct current sintering technique, often referred to as Spark Plasma Sintering (SPS), which is very effective for sintering of fully dense B_4_C at lower sintering temperatures of 1700 °C or above within 3–5 min and exhibiting good mechanical properties [45]. 

Typically, the Young’s modulus values of dense B_4_C ceramics, measured by acoustic techniques, are reported to be in the range of 460–470 GPa [46,47], however, a higher value of 570 GPa was also reported for 100% dense B_4_C [48]. The Knoop hardness of B_4_C was reported to lie in the range of 28 GPa to 31 GPa [10]. The Vickers hardness was reported to vary in a broader range of 25 to 37 GPa, depending on the composition as well as the applied load used [49,50]. The fracture toughness, measured using the length of the cracks initiated from the corners of the Vickers impressions, is not very high and reported to be in the range between 2 to 3.5 MPa m^1/2^ depending on the grain size of B_4_C [40,50]. In addition to measurements of fracture toughness using indentation techniques, the K_IC_ of B_4_C has also been measured using the single edge v-notched beam, surface crack in flexure and chevron notch beam testing techniques [51,52], where similar values were obtained. The hardness of B_4_C thin films measured by nanoindentation varied significantly in the range of 28 GPa to 60 GPa [53]. The indentation hardness of B_4_C single crystals or polycrystalline ceramics was reported to be around 39–55 GPa as measured using a Berkovich indenter [54,55,56]. Discontinuities in the loading and unloading deformation plots of B_4_C during nanoindentation were reported, which were either explained by cracking or by the transition from elastic to elastoplastic deformation caused by homogeneous nucleation of dislocations due to the high shear stresses below the indenter [55,56]. The flexural strength of B_4_C is not very high and is reported to be around 250–450 MPa [10,36,45,57,58,59], while compressive strength is much higher and lies in the range of 6.1 ± 0.3 GPa [60]. To the best of our knowledge, no measured biaxial strength data has been reported.

The thermal properties of B_4_C have been also investigated and the average reported value of the coefficient of thermal expansion is 5.73 × 10^−6^/K in the temperature range of 300–1970 K [61]. Slightly different values of coefficient of thermal expansion of B_4_C were also reported in References [62,63,64]. Boron carbide is reported to be a p-type semiconductor through its entire homogeneity range, with electronic properties dominated by hopping type transport [65,66]. The direct current (DC) electrical conductivity of boron carbide varies from 3 Ωcm to 23 Ωcm and is a function of carbon content, with a maximum in conductivity at ~13 at% C, corresponding to the B_6.5_C stoichiometry [5,67]. Here, in this research paper we present a systematic study of the properties of boron carbide sintered by Spark Plasma Sintering and also present, for the first time, biaxial strength data and compare it to 4-point bending strength data.

## 2. Materials and Methods 

**Figure 1:**
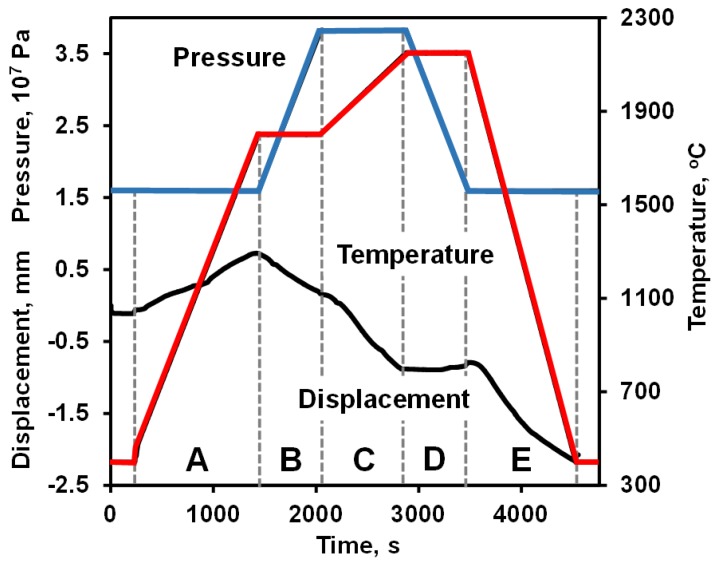
Pressure, temperature and shrinkage of B_4_C densified by SPS.

A commercially available B_4_C Grade HD07 powder from H.C. Starck (selb, Germany) was used to sinter dense B_4_C samples for flexural and biaxial testing. The 3 mm × 4 mm × 45 mm and 2 mm × 2.5 mm × 25 mm bars for bending experiments were cut from a large 100mm diameter and 10 mm thickness disk sintered in a graphite die at 2150 °C, 40 MPa with a 10 min dwell time. For the biaxial strength tests a 20 mm diameter graphite die was lined with graphite foil and charged with 2 g of B_4_C. The die was then placed into the Spark Plasma Sintering (SPS) furnace (FCTHPD25; FCTSystemeGmbH, Rauenstein, Germany) and heated at a rate of 100 °C/min under a minimum pressure of 16 MPa to 1800 °C, held for 10 min while the pressure was increased to 40 MPa and then heated at 25 °C/min to 2150 °C for a 10 min hold before cooling to room temperature over 20–25 min. The sintering regime for the B_4_C disks is shown in Figure 1. The final density of the B_4_C bars and disks after sintering was measured using Archimedes technique. 

The crystal structure of B_4_C ceramics was studied using an X-ray diffractometer (XRD, Bruker-AXS D8 Advanced Bragg-Brentano X-ray Powder Diffractometer, Bruker, WI, USA). The vibrational properties of B_4_C ceramics were studied using a Renishaw InVia Raman microscope (Renishaw Inc., Gloucestershire, UK). The Raman microscope system comprises a laser (532 nm line of solid Si) to excite the sample and a single spectrograph fitted with holographic notch filters. Before collecting spectra, the spectrometer was calibrated with a standard silicon wafer using the Si band position at 520.3 cm^−1^. The average collection time for a single spectrum was 30 s and the spectrum was collected from the polished surface of a sintered B_4_C sample. Thermal expansion measurements were carried using a thermal mechanical analyzer (NETZSCH TMA 402F3, NETZSCH-Gerätebau GmbH, Selb, in Germany) in the temperature range of 30 to 1000 °C with a heating rate of 5 °C/min. One bar of B_4_C with dimensions of 3 mm × 4 mm × 25 mm was used for the measurement of thermal expansion. The load was set to 0.1 N and the average linear coefficient of thermal expansion was determined from the obtained thermal expansion data. Room temperature DC electrical conductivity of the 4 mm × 3 mm × 45 mm bars was measured using a 4-probe experimental set up connected to a Keithley 2450 source meter (Textronix UK, Ltd., Berkshire, UK). Four B_4_C samples were used for the measurements of electrical conductivity. A potential difference of 1 V was applied through the outer probes and the corresponding current between the inner probes was measured for calculation of the resistance values. 

The Impulse Excitation Technique (IE, Grindo-SonicMk5“Industrial” J.W. Lemmens, Leuven, Belgium) was used to determine the elastic modulus of the B_4_C bars at room temperature and the measurements were carried out in accordance with the EN843-2 standard [68]. Samples, in the form of 3 mm × 4 mm × 45 mm bars of known density, were lined up with a supporting cylinder and placed over a microphone. To determine the elastic modulus at room temperature using the natural frequency of vibration of the bars, they were struck lightly using a small hammer and the acoustic vibrations were recorded using the microphone. Then, using the dimensions of the sample, its density and natural frequency of vibration, the Young’s modulus was calculated. A total of four samples were used for the IE measurements.

In addition to the IE measurement, the Young’s and shear moduli of B_4_C were also measured using a Resonant Ultrasound Spectroscopy technique using a custom made high-temperature resonant ultrasound spectroscope (HT-RUS) that utilizes a commercially available room temperature (RT) RUS (Magnaflux Quasar, Albuquerque, NM, USA) system. RUS is a high-precision dynamic technique, which is used to determine the elastic moduli and energy dissipation (mechanical damping) of materials by measuring the vibrational spectrum of samples with well-defined geometry, usually in the shape of parallelepipeds or cylinders [69,70,71,72,73]. A B_4_C sample in the form of disc with 20 mm diameter and 1.5 mm thickness was supported by three piezoelectric transducers. One transducer, which is a transmitting transducer, generates an elastic wave of constant amplitude but of varying frequency covering a large number of vibrational eigenmodes of the sample. The resonance response of the excited sample is detected by the other two transducers, which are receiving transducers. To study the elastic moduli as a function of temperature, SiC extension rods were added to transmit the ultrasound waves to the RT RUS equipment. This arrangement allowed the B_4_C disk to be held on the tip of the extension rods, at the desired temperature in the furnace, while the transducers were unaffected by high temperature. The measurements were performed under vacuum. The B_4_C sample was heated at a rate of 10 °C/min and resonance spectra were collected at an interval of 25 °C up to 1000 °C after an isothermal hold of 20 min. Depending on the density and stiffness of the material, measurements were done in the 20–500 kHz frequency range to cover the first 40 frequencies. The RUS spectra cannot be de-convoluted directly to deduce the elastic constants. Starting from the known sample dimensions, density and a set of “guessed” elastic constants—namely C_11_ and C_44_ for an isotropic solid, where C_11_ = 542.8 GPa and C_44_ = 164.8 GPa as reported in Reference [21], the elastic moduli were determined from collected RUS spectra using a multidimensional algorithm (MagnafluxQuasar, Albuquerque, NM, USA) that minimizes the root-mean-square (RMS) error between the measured and calculated resonant peaks. Two B_4_C samples were used for the measurements of elastic properties by RUS.

Nanoindentation of B_4_C was performed using a Hysitron TI Premier machine equipped with a Berkovich tip. A total of 25 indents were produced on the polished surface of B_4_C samples using a maximum load of 9500 μN, which was held for 3s at the maximum load between loading and unloading. Load vs. displacement curves were analyzed to calculate the hardness, *H* and reduced modulus, *E_r_*, using a method described by Oliver and Pharr [74,75]. 

The mean contact pressure was calculated as

(1) $$p_{i}=\frac{P_{i}}{A_{i}},$$

where *P_i_* is the instantaneous indentation load taken from the corresponding load—displacement diagram (*P_i_* may be taken directly from indentation data points); *A_i_* is the contact area, which is determined using the area function:

(2) $$A_{i}=C_{0}{\left(h_{c}\right)}_{i}^{2}+C_{1}{\left(h_{c}\right)}_{i}+C_{2}{\left(h_{c}\right)}_{i}^{1/2}+C_{3}{\left(h_{c}\right)}_{i}^{1/4}+C_{4}{\left(h_{c}\right)}_{i}^{1/8}+C_{5}{\left(h_{c}\right)}_{i}^{1/16},$$

where $\;C_{0}$, $C_{1}$, $C_{2}$, $C_{3}$, $C_{4}$ and $C_{5}$ are the coefficients determined for a given indenter from a series of indents at various contact depths in a sample of known elastic modulus (typically fused quartz). Note that $C_{0}$ = 24.5 for an ideal Berkovich probe; ${\left(h_{c}\right)}_{i}$ is the contact depth corresponding to $P_{i}$, which can be calculated as

(3) $${\left(h_{c}\right)}_{i}=h_{i}-{\left(h_{e}\right)}_{i},$$

where $h_{i}$ is the total measured indenter displacement corresponding to $P_{i}$ ($h_{i}$ may be taken directly from indentation data points); ${\left(h_{e}\right)}_{i}$ is the corresponding elastic deflection [74,75,76,77,78]:

(4) $${\left(h_{e}\right)}_{i}={\left(h_{e}\right)}_{\max}\sqrt{\frac{P_{i}}{P_{\max}}}=\varepsilon \frac{P_{\max}}{S}\sqrt{\frac{P_{i}}{P_{\max}}},$$

where ${\left(h_{e}\right)}_{\max}$ is the elastic deflection at maximum load $P_{\max}$ of the indentation diagram; the unloading stiffness *S* is the slope at the beginning of the unloading portion of the indentation diagram; is a constant that depends on the indenter’s geometry (ε = 0.75 for Berkovich indenter). After calculation, the mean contact pressure can be plotted against the contact depth.

The B_4_C sample surface was first ground and further polished down to a 1 mm diamond grit size to determine the Vickers hardness of the ceramics. A Vickers hardness tester Durimet (Ernst-Leitz, Germany) was used for hardness tests in accordance with EN843-4 standard. The hardness of the samples was measured using a 9.8 N load applied for a period of 15 s. Twenty impressions were produced for the measurements of the sizes of the impression diagonals. The hardness *H* (GPa) was calculated according to the equation [79].

(5) $$H=1854\;\frac{P}{d^{2}},$$

where *P* is the indentation load in N and d is the impression diagonal length in µm.

Four-point bending tests were performed to measure flexure strength using B_4_C samples with dimensions of 2 mm × 2.5 mm × 25 mm. Seven B_4_C samples were used to measure the flexural strength. To obtain load-displacement bending diagrams, the B_4_C samples were loaded using a four-point bending jig with 3 mm diameter rollers with a 10 mm loading span and 20 mm supporting span using a 2 kN load cell on an universal testing machine (Zwick, Ulm, Germany) in accordance with the EN 843-1 standard [80]. 

The biaxial strength of B_4_C samples were tested using a ring-on-ring testing jig [81]. A total of 27 B_4_C samples were used for the biaxial strength testing. Disks with 20 mm diameter and 1.5 mm thickness were placed on the support ring and loaded with a loading ring in load control mode with a loading rate of 80 N/s or 22 MPa/s until failure of the disk occurred. The disks were polished on one side and the polished surface was on the tensile side of the ring-on-ring fixture. The recorded load was recalculated into stress using the ASTM Standard C1499 equation [82]. As the deflection of the disk was not recorded during loading, the deformation of the B_4_C samples was calculated using the measured Young’s modulus in the elastic beam equation, as it is well known that B_4_C behave elastically and does not show any plasticity during static deformation at room temperature [83,84]. A standard Weibull analysis of strength data was carried out [85,86,87,88].

Finite Elements Analysis (FEA) modeling of the biaxial strength testing of B_4_C was performed using the functions of the Simulia Abaqus^®^ 6.11-1 software (Dassault Systems, Vélizy-Villacoublay, Paris, France). The B_4_C specimen was treated as a deformable body and the loading/supporting rings as discrete rigid bodies. Load and support rings were defined as a 2D axisymmetric and homogenous model, with properties of 4140 alloy steel. The sample was defined using properties t obtained experimentally from the B_4_C biaxial strength tests. The elastic modulus and the Poisson’s ratio for a B_4_C sample were taken as 429 GPa and 0.185 and for 4140 alloy steel were taken as 200 GPa and 0.29, respectively. The contact between the specimen and ring-on-ring surfaces were defined as surface-to-surface contacts with a master surface on the rings. The simulation was performed with a friction of 0.1. In order to conduct a simulation, a support-ring was fully fixed in its position in any direction, however the loading ring was fully fixed besides the direction of the force in the Y-axis. The experimental data showed that the sample broken at a load of 1530 N, therefore the force for the simulation for the loading ring was chosen to be 1530 N in order to simulate a uniform pressure along the load-ring surface area. The mesh element type was defined as Discrete Rigid Element (RAX2) and for the sample as an Axisymmetric Stress (CAX4) with a reduced integration. The size of the mesh for a sample was chosen to be 0.2 mm and 0.1 mm for the loading and supporting rings. 

Fracture toughness was measured using the Single Edge V Notch Beam (SEVNB) technique in accordance with the CEN/TS 14425-5 standard [89]. Three B_4_C samples were used for the K_1C_ testing. A single notch was made on the 3 mm side of the 3 × 4 × 45 mm^3^ bar as near to the center as possible with a depth between 20% and 40% of the total thickness of the bar, since it was shown that within this range the depth of the notch has no influence on the measured K_1c_ values [90]. A diamond saw was used to make the initial 0.5 mm depth notch; after that the final 1–1.5 mm depth notch with ∼1.8-2.2 μm tip radius was produced by machine cutting using a razor blade with 6 μm and then 1 μm diamond paste deposited. Three samples were tested at room temperature with a crosshead speed of 0.5 mm/min. 

## 3. Results and Discussions 

### 3.1. Shrinkage Behavior, Structure, Thermal Expansion and Electrical Conductivity of Sintered B_4_C Ceramics

Figure 1 shows the shrinkage of B_4_C ceramic during Spark Plasma Sintering together with the pressure and temperature profiles, as a function of sintering time. The sample was heated from 400 °C to 1800 °C in 20 min under a minimal pressure of 16 MPa, then dwelled for 10 min during which a pressure of 40 MPa was fully applied, then the temperature was increased to 2150 °C and dwelled at this sintering temperature for 10 min before cooling down to room temperature. To protect the sample from fracture, the 40 MPa pressure was decreased at the beginning of the dwell time at 2150 °C in a such way that it reduced back to 16 MPa in 10 min by the end of dwell. As can be seen in Figure 1, expansion of the equipment was observed upon heating all of the way up to 1800 °C (region A, Figure 1) until the moment when the applied pressure was increased, causing shrinkage of the sample (region B, Figure 1). Under the constant pressure but with temperature increasing, most of the shrinkage of the B_4_C sample occurred (section C, Figure 1). When the sample dwelled at 2150 °C but with decreasing pressure, the shrinkage remained constant (section D, Figure 1), possibly because the majority of the densification process had already occurred. During cooling from the sintering temperature, shrinkage of the sample occurred (section E, Figure 1). After sintering, the samples were machined, surfaces polished and the density was measured to be equal to 2.50 ± 0.07 g/cm^3^ showing less than 1% of porosity.

**Figure 2:**
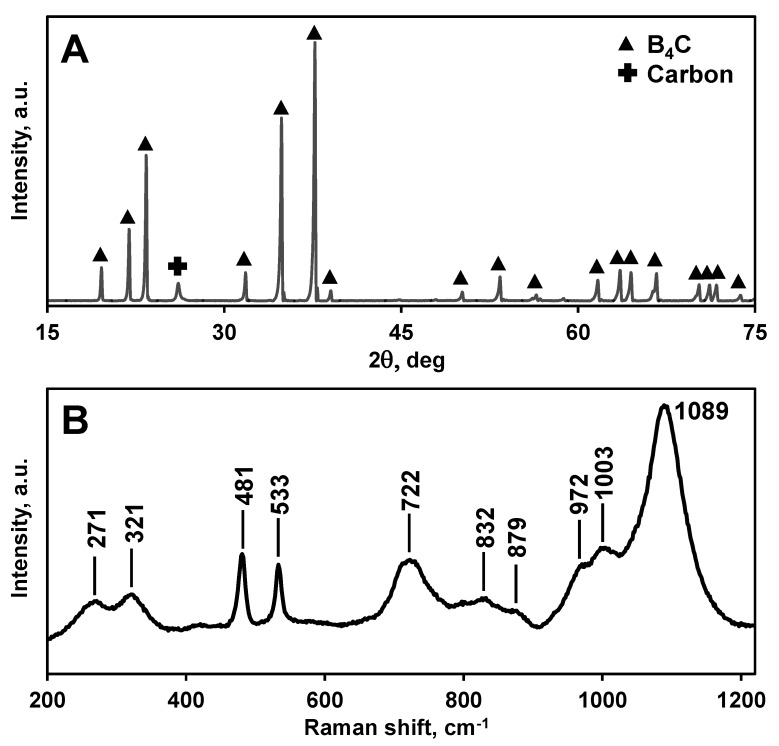
(**A**) X-ray diffraction pattern and (**B**) Raman spectrum of B_4_C taken from polished surface.

**Figure 3:**
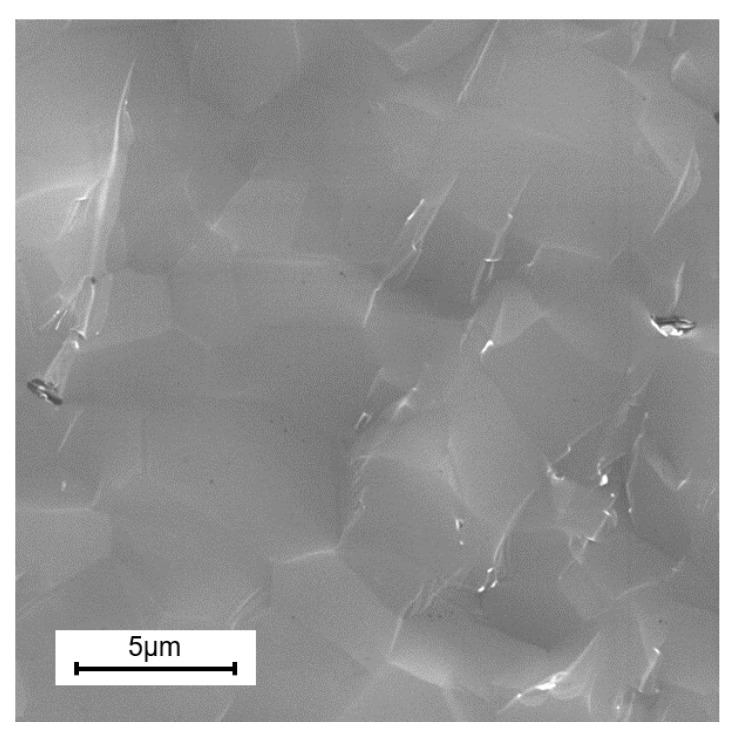
Scanning electron micrograph (SEM) of fracture surface of B_4_C after flexture strength testing.

An X-ray diffraction pattern taken from polished surface of B_4_C is shown in Figure 2A. All of the diffraction peaks in the pattern belong to the rhombohedral Bravais lattice of space group of B_4_C and the peak at ~26.16° 2ϴ belonging to carbon indicates that a small quantity of carbon was present as a secondary phase [8,91]. Raman spectrum obtained using a 532 nm laser measured from a polished surface of B_4_C is shown in Figure 2B. The spectrum resembles the surface spectrum of B_4_C published in Reference [91,92]. The grain size of B_4_C was equal to 3.4 ± 0.05 micron as estimated from the fracture surface of broken samples (Figure 3). 

**Figure 4:**
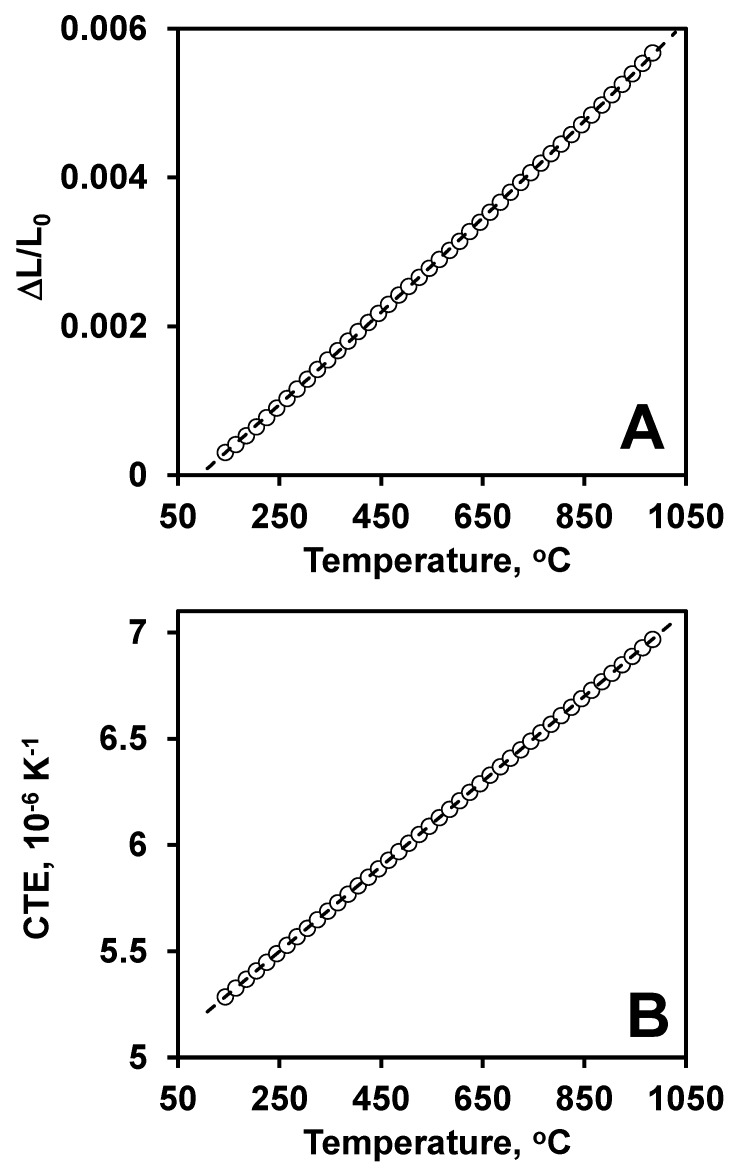
(**A**) Thermal expansion and (**B**) coefficient of thermal expansion of B_4_C as a function of temperature.

The thermal expansion of B_4_C was almost linear and the average coefficient of thermal expansion was calculated to be equal to 6 × 10^−6^ / K in the 25–1000 °C temperature range (Figure 4). The measured values of CTE corresponded very well to previously published results, where the CTE of hot pressed B_4_C was reported to be equal to 6 × 10^−6^ /K [93]. The room temperature DC electrical conductivity was measured to be equal to 0.00284 ± 0.0009 Ω m (Table 1), which is typical of the values reported for B_4_C [63]. 

### 3.2. Elastic Properties of B_4_C by Impulse Excitation (IE) Technique and Resonant Ultrasound Spectroscopy

The Young’s modulus of B_4_C measured using the IE technique at room temperature was equal to 442 ± 3 GPa (Table 1), which corresponds very well with the values of 440 to 560 GPa reported in other papers [94,95]. 

**Figure 5:**
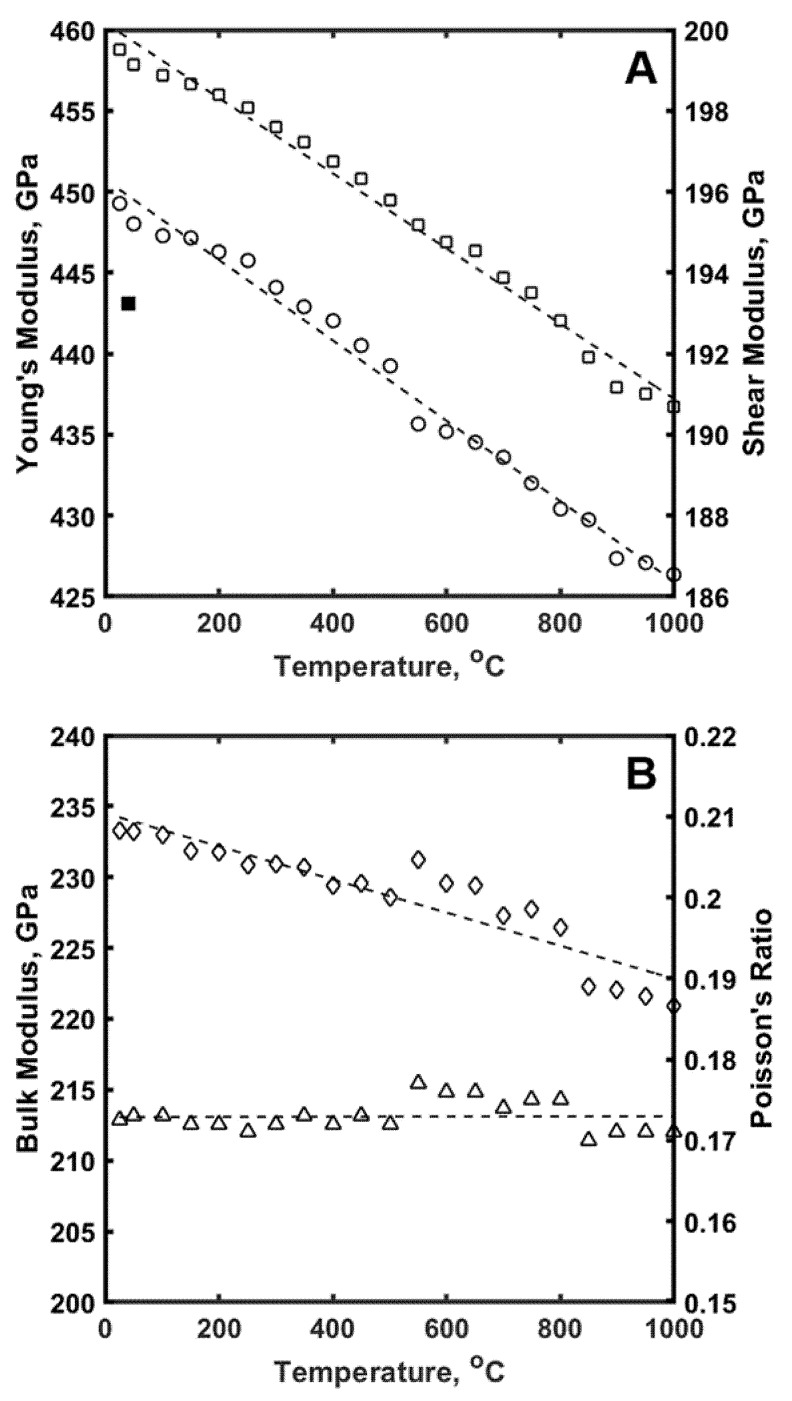
Elastic properties of B_4_C measured by Resonance Ultrasound Spectroscopy (RUS). (**A**) Young’s modulus (□) and Shear modulus (○) as a function of temperature: (**B**) Bulk modulus (◊) and Poisson’s ratio (∆) as a function of temperature. Young’s modulus (■) measured by IE.

The results of the measurements of Young’s and the shear moduli along with bulk modulus and Poisson’s ratio by RUS as a function of temperature are shown in the Figure 5. The Young’s modulus of B_4_C measured by RUS was slightly higher compared to that measured using IE and was equal to 458.7 GPa at room temperature. The Young’s modulus decreased linearly upon heating in in vacuum and was 436.7 at 1000 °C. The shear modulus, also measured by RUS, was equal to 195.7 GPa at room temperature but slightly decreased linearly on heating to 186.5 GPa at 1000 °C. The bulk modulus and Poisson’s ratio were calculated from Young’s and shear moduli data and were equal to 220.9 GPa and 0.172 at 1000 °C. Note that Poisson’s ratio of B_4_C remains the same for the whole RT –1000 °C temperature interval (Figure 5B). All of the values of elastic properties of B_4_C presented in Figure 5 correspond very well with previously published data, where Young modulus was reported in the range of 440–560 GPa, shear modulus was reported in the range of 188–194 GPa, bulk modulus was reported in the range of 220–248 GPa and Poisson’s ratio was reported in the range 0.19–0.21 [94,95,96,97].

### 3.3. Young’s Modulus and Hardness by Nanoindentation and Vickers Hardness

**Figure 6:**
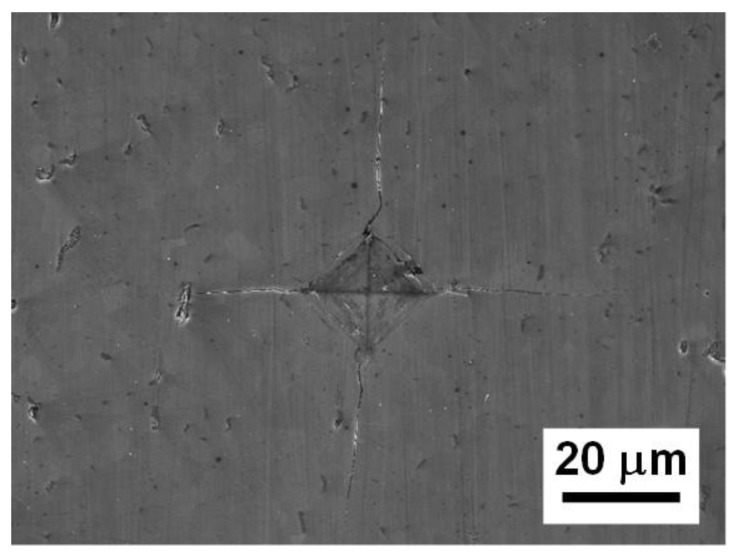
SEM micrograph of Vickers hardness impression in B_4_C made at 1kg indentation load.

The Young’s modulus along with hardness of B_4_C was also measured using the nanoindentation technique. A total 50 impressions were made into a polished B_4_C surface using a Berkovich indenter and the average values of Young’s modulus and hardness were measured to be equal to 419.2 ± 47.3 GPa and 41.1 ± 5.7 GPa, respectively (Table 1). The Young’s modulus values measured by nanoindentation are very similar to the ones measured by IE and RUS but were slightly lower than the 506 GPa values, also measured by nanoindentation, reported in Reference [98]. However, in Reference [98] the reported high value of 506 GPa for the Young’s modulus was measured on a single crystal in one specific crystallographic directions of (0001) and (10−11) respectively, thus the anisotropy of the bond strength in a certain crystallographic direction of B_4_C may explain this discrepancy. The average hardness value measured during nanoindentation was calculated to be equal to 41.1 ± 5.7 GPa, while the Vickers hardness measured using a microhardness tester was equal to 28.5 ± 1.2 GPa (Table 1). The Vickers hardness impression after indention with a 1 kg load is shown in Figure 6.

**Figure 7:**
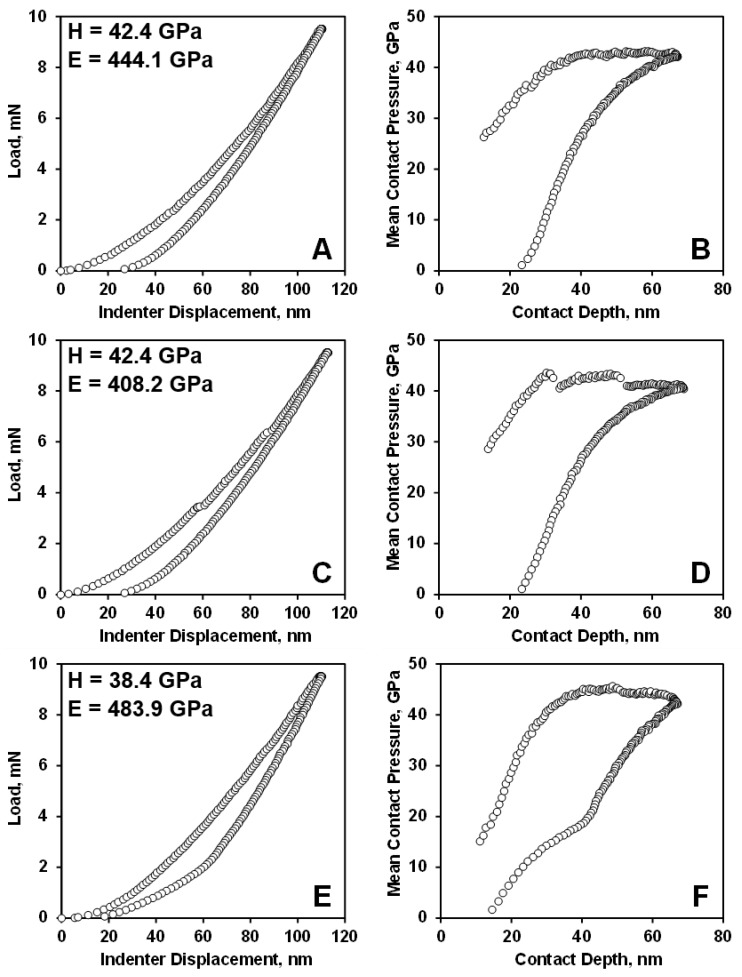
Nanoindentation load-displacement curves (**A**,**C**,**E**) and mean contact pressure-contact depths (**B**,**D**,**F**) for B_4_C for loading and unloading indentation behavior.

The three different load-displacement plots in Figure 7 illustrate the different types of nanoindentation behavior observed. While many of the measured load-displacement plots were smooth and showed no deviation from a continuous increase in load and displacement (Figure 7A), many of the load-displacement plots showed multiple or at least one pop-in events upon loading (Figure 7C). While only three load-displacement plots in the data set of 50 indentation plots that showed a well pronounced “elbow” effect upon unloading (Figure 7E). The absence or presence of “pop-ins” or “elbows” in the load-displacement plots during loading and unloading of B_4_C are indicative of the absence or presence of structural changes such as crystal phase transitions or amorphization. It is generally accepted that the absence of sudden volumetric changes associated with a structural transformation in the material produce a monotonic loading/unloading response during nanoindentation (Figure 7A) [5]. 

The maximum mean contact pressure under such conditions is about 43 GPa (Figure 7B), which corresponds very well to the average hardness values of 41.1 ± 5.7 GPa (Table 1). Some of the load-displacement plots obtained during nanoindentation of B_4_C exhibited one to three discontinuities during loading (Figure 7C). The presence of such pop-in events during nanoindentation is explained by transition from the elastic to elastoplastic deformation upon nanocontact in the imprint [55]. A second order phase transition in B_4_C was reported to occur at 32 GPa to 35 GPa due to reordering of polar atoms [99]. It was found that this second order transition, which is characterized by atomic site exchange with hysteresis, is a relaxation process that is reversible [100]. It was also predicted that both the chain bending of the three-atom carbon chain and disordering of the structure were detected above 70 GPa, where non-hydrostatic stresses are present. It was explained that the non-ideal structure of B_4_C can activate chain bending guided by polar carbon atom location in the icosahedron even at lower stresses above 38 GPa as observed in shock-wave experiments [101]. The discontinuities during nanoindentation of B_4_C occurred first at 43 GPa with a mean contact pressure decreasing to below 40 GPa during this first pop-in event occurring at a contact depth of 20 nm (Figure 7D). 

Upon further loading, the mean contact pressure recovered back to above 40 GPa, where a second pop-in event was detected at a contact penetration depth of about 50 nm. The appearance of such pop-in events during loading of B_4_C can possibly be explained either by plastic deformation by nucleation of dislocations [55] or a high pressure structural phase transition, with an associated decrease in the volume of the high-pressure phase [99,101]. The simulated estimate of ~4% sudden volume reduction were reported in [102], however, the pressure where such a significant volume change would occur was estimated to be ~22.8 GPa. Structural recovery and formation of disordered phases were reported to occur in a number of materials upon unloading [103]. The discontinuities and changes in the slopes upon unloading of B_4_C were reported in [56]. In our nanoindentation experiments, three load-displacement nanoindentation plots exhibited the formation of an “elbow” (Figure 7E), which can be explained by the amorphization of the deformed B_4_C structure upon unloading and the formation of a phase with larger volume, causing a change in the slope of the mean contact pressure vs. contact depth deformation plot (Figure 7F).

### 3.4. Strength and Fracture Toughness of B_4_C 

**Figure 8:**
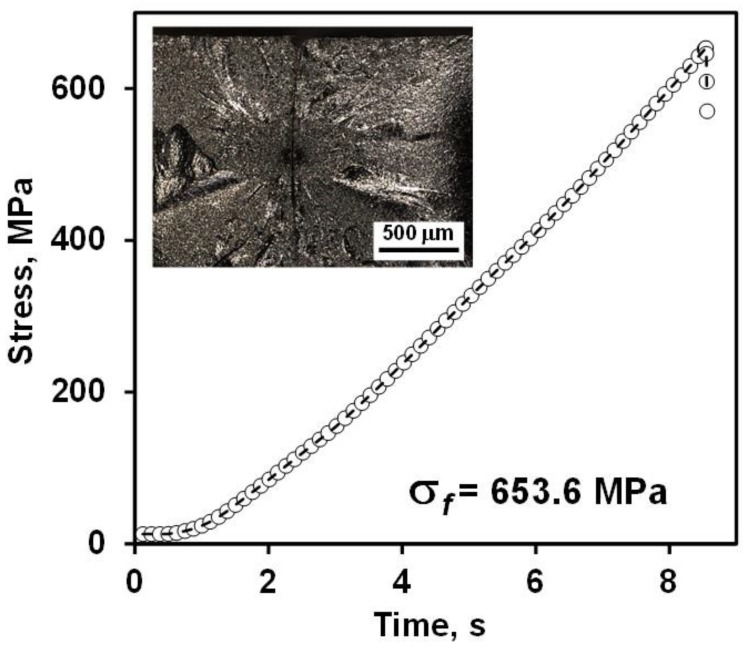
Stress vs. time loading plot of B_4_C for flexure strength measurements. The insert shows an optical micrographs of fracture surface of B_4_C after failure.

While B_4_C exhibits a very high Young’s modulus and hardness compared to other ceramics, its average flexural strength is not so highs and it averages between 250–450 MPa [10,36,41,45,57,58,59,104,105,106]. The flexural strength of B_4_C measured in the current research had an average value of 585 ± 70 MPa. The relatively high flexural strength in the current study suggests that the strength determining defects must have been relatively small. A typical stress vs. time plot of loading of B_4_C in 4-point bending is shown in Figure 8, with inserts showing the fracture surface of B_4_C samples after failure.

**Figure 9:**
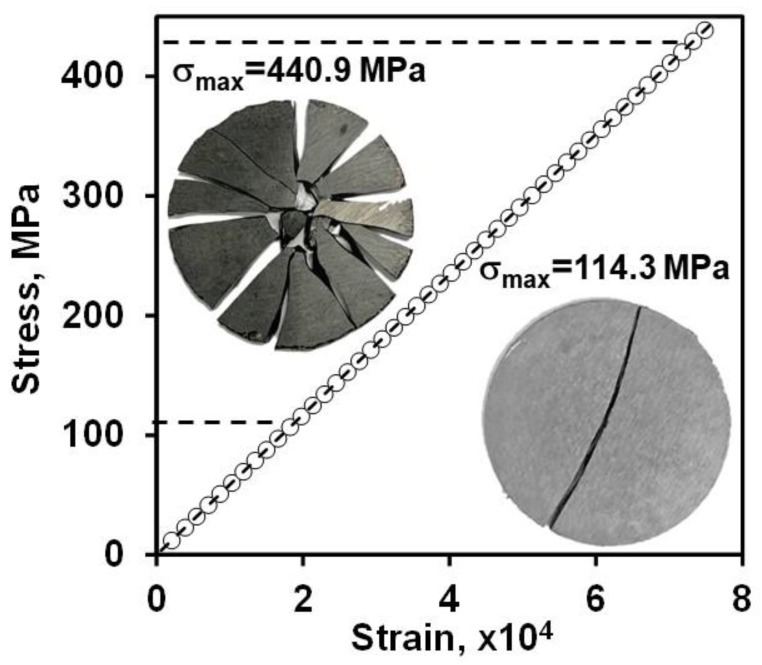
Biaxial stress-strain deformation plot and two photos of B_4_C samples that failed at the maximum and the minimum biaxial stress applied.

The average biaxial strength *σ_b_* of B_4_C was measured to be 239 ± 122 MPa using a ring-on-ring configuration [81]. The typical stress-strain deformation behavior of B_4_C during ring-on-ring loading is shown in Figure 9. The two inserts show micrographs of B_4_C samples after failure. As expected, the samples that failed at relatively high loads were broken into multiple pieces after failure, while the samples that failed at low loads were broken into two pieces, as can be seen in the inserts Figure 9. It is not clear why the strength of B_4_C measured in 4-point bending and ring-on-ring tests were so different but the quality of the surface after machining likely to contributed to such low biaxial strength values.

**Figure 10:**
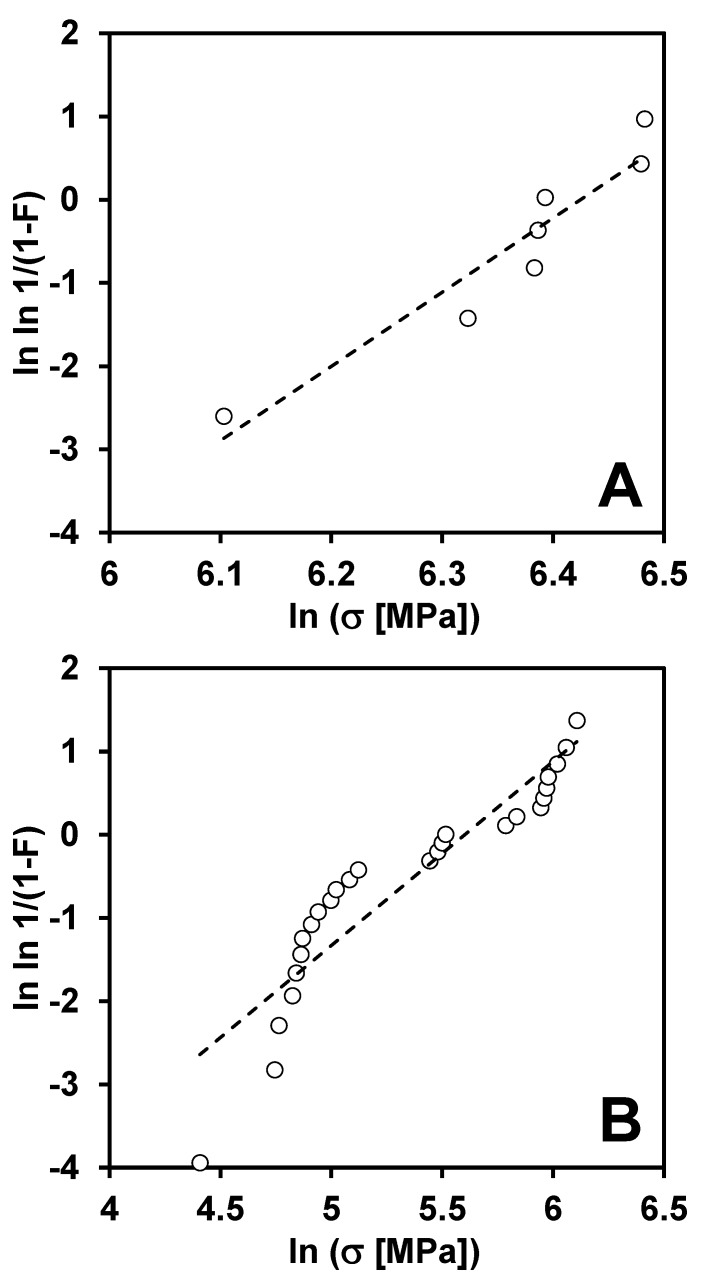
The Weibull probability plots of (**A**) 4-point flexure strength and (**B**) ring-on-ring biaxial strength.

Weibull modulus m_f_ is equal to 9.9 for 4-point bending strength values but the Weibull modulus m_b_ is only 2.2 for the ring-on-ring strength values (Table 1). The Weibull distribution for biaxial as well as 4-point bending is presented in the Figure 10. The characteristic strength σ_0_f was equal to 611 MPa in 4-point bending experiments and the scale parameter σ_0b_ was equal to 271 MPa in ring-on-ring tests (Table 1). 

**Figure 11:**
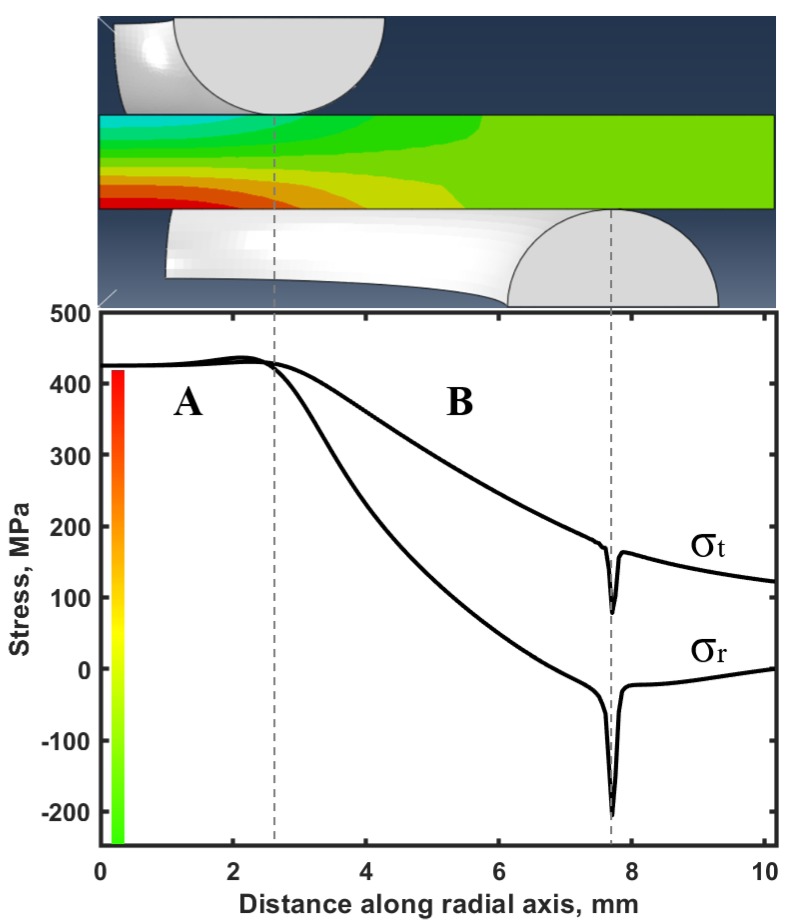
The stress distribution in a B_4_C disk upon biaxial loading. σr is the stress distribution in the radial direction and σt is the stress distribution in the tangential direction.

The maximum biaxial strength during the mechanical testing of B_4_C specimens came out to be 440 MPa with a maximum applied load of 1530 N, which was used for a numerical simulation. The stress distribution during biaxial ring-on-ring loading of B_4_C was modeled using Abaqus [81], which showed that the highest tensile biaxial strength occurred along the bottom surface of B_4_C specimen (Figure 11) and was equal to 436.1 MPa. The region A in Figure 11 represents the highest uniform tensile stresses at the polished bottom surface of B_4_C between the surface contact of the specimen and support-ring, as well as a high compressive strength which developed at the line of the contact of the B_4_C samples with the load-ring. 

**Figure 12:**
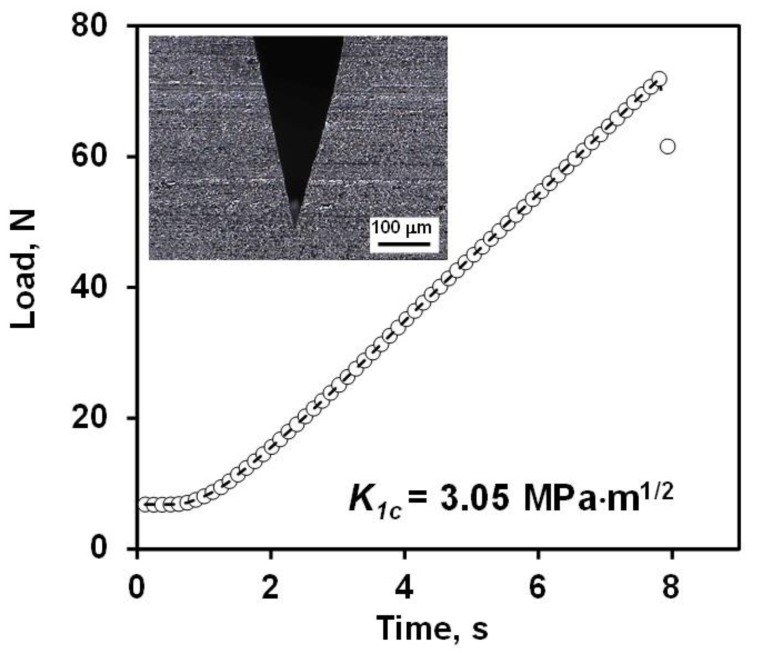
Load vs. time plot of B_4_C V notched bar used for the calculation of fracture toughness. The insert is an optical micrograph of the V notch.

The region B in the Figure 11 depicts the stresses that are no longer uniform and by the end of region B, at the point when the sample has a contact with the support ring, the compressive stresses rise rapidly. However, it does not represent the critical condition and the values show that it is not a critical point of failure during testing. The stress distribution depicted by a numerical model are similar to those reported in a previous study of the biaxial strength of ZrB_2_- SiB_6_ ceramic composite [81]. The fracture toughness of B_4_C measured by SEVNB was equal to the 3 ± 0.19 MPa m^1/2^. The load vs. time plot used for the calculation of K_lC_ is shown in Figure 12, while the insert shows an optical micrograph of the V-notch with the measured tip diameter equal to 2.1 µm.

## 4. Conclusions

The thermal, electrical and mechanical properties of dense B_4_C ceramics (99%) sintered using Spark Plasma Sintering were investigated. It was determined by XRD and Raman spectroscopy that the major phase was indeed B_4_C. A minor presence of a C phase was also detected by X-ray diffraction. The grain size of B_4_C after sintering was in the range of 2.5–3.5 micron as estimated by SEM. Both the measured thermal expansion and electrical conductivity of the B_4_C ceramics is similar to data published in the literature. The Young’s modulus of B_4_C measured by three different techniques—IE, RUS and nanoindentation showed a very good overlap in values, which ranges from 419.2 ± 47.3 GPa for nanoindentation to 458.7 GPa for RUS measurements at room temperature. Both the Young’s, shear and bulk moduli decreased by about ~5% at 1000 °C compared to their room temperature values, however, the Poisson’s ratio remained constant at 0.172 in the whole RT to 1000 °C temperature range measured by RUS. The difference in hardness values measured by nanoindentation as 41.1 ± 5.7 GPa and Vickers microhardness at 1kg as 28.5 ± 1.2 GPa was expected and it could be explained by indentation size effect and/or formation of radial cracks from the corner of impressions, which relieved the indentation stress and decreased the hardness value above a certain critical load during indentation. The mean contact pressure-contact depth plots obtained from load-displacement nanoindentation data indicated pop-in events during loading and an “elbow” event during unloading, both of which are indicative of possible structural changes in B_4_C structure during nanoindentation. The 4-point bending strength of the B_4_C ceramics was σ_o_ 585 ± 70 MPa with a shape parameter mf equal to 9.9 and scale parameter σ_of_ equal to 611 MPa. The biaxial strength pf B_4_C was measured to be much lower and equal to 238.6 ± 122 MPa with a shape parameter of 2.2 and scale parameter σ_ob_ equal to 271 MPa. It was determined that failure occurred by fully transgranular fracture, with no intergranular failure. Using the SEVNB technique, a K_lc_ = 3 ± 0.19 was measured for B_4_C, which is similar to previously reported values. 

## Figures and Tables

**Table 1:** Thermal, electrical and mechanical properties of B_4_C at room temperature.

CTE 10^−6^ K^−1^ (Dilatometer)	6
Ω, Om m	2.84 × 10^−3^ ± 0.9
*E*, GPa (IE)	442 ± 3
*E*, GPa (RUS)	458.7
*G*, GPa (RUS)	195.7
*K*, GPa (RUS)	233.3
ν (RUS)	0.173
*E*, GPa (Nanoindentation)	419.2 ± 47.3
*H*, GPa (Nanoindentation)	41.1 ± 5.7
*H_V_*, GPa (Vickers)	28.5 ± 1.2
*σ_f_*, MPa (Flexure)	585 ± 70
*m_f_* (Flexure)	9.9
*σ*_0*f*_, MPa (Flexure)	611
*σ_b_*, MPa (Biaxial)	238.6 ± 122
*m_b_* (Biaxial)	2.2
*σ*_0*b*_, MPa (Biaxial)	271
*K_1c_*, MPa m^1/2^(SEVNB)	3 ± 0.19

## Abbreviation

IE	Impulse Excitation Technique
RUS	Resonant Ultrasound Spectroscopy
CTE	coefficient of thermal expansion
SEVNB	Single Edge V Notch Beam Technique
RT	room temperature
Ω	Electrical conductivity
E, (IE)	Young’s modulus measured by IE
E, (RUS)	Young’s modulus measured by RUS
G, (RUS)	Shear modulus measured by RUS
K, (RUS)	Bulk modulus measured by RUS
ν (RUS)	Poisson’s ration measured by RUS
E, (Nanoindentation)	Young’s, modulus measured by nanoindentation technique
H, (Nanoindentation)	Hardness measured by nanoindentation technique
H_V_ (Vickers)	Vickers hardness
σ_f_ (Flexure)	Flexural strength measured by 4-point bending
m_f_ (Flexure)	Weibull modulus measured by 4-point bending
σ_0f_ (Flexure)	Scale parameter for 4-point bending
σ_b_ (Biaxial)	Biaxial strength for ring-on-ring test
m_b_ (Biaxial)	Weibull modulus for biaxial strength testing
σ_0b_ (Biaxial)	Scale parameter for biaxial strength testing
K_1C_ (SEVNB)	Fracture toughness measured by Single Edge V Notch Beam Technique
